# Supplementary material for: Microneedle manipulation of the mammalian spindle reveals specialized, short-lived reinforcement near chromosomes
Source: eLife. 2020 Mar 19;9:e53807. doi: 10.7554/eLife.53807 (PMC7117910; doi:10.7554/eLife.53807)
Supplement: Transparent reporting form [file elife-53807-transrepform.docx]

***eLife’s* transparent reporting form**

We encourage authors to provide detailed information *within their submission* to facilitate the interpretation and replication of experiments. Authors can upload supporting documentation to indicate the use of appropriate reporting guidelines for health-related research (see [EQUATOR Network](http://www.equator-network.org/%20)), life science research (see the [BioSharing Information Resource](https://biosharing.org/)), or the [ARRIVE guidelines](http://www.plosbiology.org/article/info:doi/10.1371/journal.pbio.1000412) for reporting work involving animal research. Where applicable, authors should refer to any relevant reporting standards documents in this form.

If you have any questions, please consult our Journal Policies and/or contact us: [editorial@elifesciences.org](mailto:editorial@elifesciences.org).

**Sample-size estimation**

- You should state whether an appropriate sample size was computed when the study was being designed
- You should state the statistical method of sample size computation and any required assumptions
- If no explicit power analysis was used, you should describe how you decided what sample (replicate) size (number) to use

Please outline where this information can be found within the submission (e.g., sections or figure legends), or explain why this information doesn’t apply to your submission:

For each experiment, sample size is presented in the figure legend and was chosen based on standards in the field and experimental limitations and variability.

**Replicates**

- You should report how often each experiment was performed
- You should include a definition of biological versus technical replication
- The data obtained should be provided and sufficient information should be provided to indicate the number of independent biological and/or technical replicates
- If you encountered any outliers, you should describe how these were handled
- Criteria for exclusion/inclusion of data should be clearly stated
- High-throughput sequence data should be uploaded before submission, with a private link for reviewers provided (these are available from both GEO and ArrayExpress)

Please outline where this information can be found within the submission (e.g., sections or figure legends), or explain why this information doesn’t apply to your submission:

The number of replicates for each experiment that was performed is presented in the related figure legends. We discuss our definition of biological replication in the Statistics section of the Materials and Methods, and we provide sufficient information in our legends to understand the number of each type of replicate performed for a given experiment.

We explain our criteria for inclusion or exclusion of data in the Materials and Methods section. e.g., “K-fibers were included in the data set (Figure 2) only if their entire length stayed within the same z-plane over time”.

**Statistical reporting**

- Statistical analysis methods should be described and justified
- Raw data should be presented in figures whenever informative to do so (typically when N per group is less than 10)
- For each experiment, you should identify the statistical tests used, exact values of N, definitions of center, methods of multiple test correction, and dispersion and precision measures (e.g., mean, median, SD, SEM, confidence intervals; and, for the major substantive results, a measure of effect size (e.g., Pearson's r, Cohen's d)
- Report exact p-values wherever possible alongside the summary statistics and 95% confidence intervals. These should be reported for all key questions and not only when the p-value is less than 0.05.

Please outline where this information can be found within the submission (e.g., sections or figure legends), or explain why this information doesn’t apply to your submission:

Statistical analysis methods and types of tests are described in the Statistical tests section of the Materials and Methods. For each experiment, we describe exact values of N, dispersion/precision measures (mean, SEM), effect size (Spearman R), p-values and type of statistical test conducted in the Figure Legends.

(For large datasets, or papers with a very large number of statistical tests, you may upload a single table file with tests, Ns, etc., with reference to sections in the manuscript.)

**Group allocation**

- Indicate how samples were allocated into experimental groups (in the case of clinical studies, please specify allocation to treatment method); if randomization was used, please also state if restricted randomization was applied
- Indicate if masking was used during group allocation, data collection and/or data analysis

Please outline where this information can be found within the submission (e.g., sections or figure legends), or explain why this information doesn’t apply to your submission:

Datasets were grouped by experimental conditions as indicated in the text and figure legends. Experimental conditions include type of mechanical perturbation and molecular background.

**Additional data files (“source data”)**

- We encourage you to upload relevant additional data files, such as numerical data that are represented as a graph in a figure, or as a summary table
- Where provided, these should be in the most useful format, and they can be uploaded as “Source data” files linked to a main figure or table
- Include model definition files including the full list of parameters used
- Include code used for data analysis (e.g., R, MatLab)
- Avoid stating that data files are “available upon request”

Please indicate the figures or tables for which source data files have been provided:

Source data has been provided for the following figures and supplentary figures: Figure 1C, Figure 1F, Figure 2D, Figure 2F, Figure 2G, Figure 2H, Figure 3C, Figure 3F, Figure 3G, Figure 3I, Figure 4B, Figure 4E, Figure 4F, Figure 4H, Figure 4I, Figure 4J and Figure 2- figure supplement 1, Figure 2- figure supplement 3, Figure 2- figure supplement 4, Figure 3- figure supplement 1, Figure 3- figure supplement 2, Figure 3- figure supplement 3, Figure 4- figure supplement 1C, Figure 3- figure supplement 2.
